# Evaluation of Aqueous Maceration and Ultrasound-Assisted Extracts of *Physalis philadelphica* Lam. Solanaceae Husk on Hyperglycemia, Insulin Resistance, Hepatic Steatosis, and Oxidative Stress Markers in Obese Rats

**DOI:** 10.3390/ph18111655

**Published:** 2025-11-01

**Authors:** Juliana Morales-Castro, Jazel Barragán-Zúñiga, María Inés Guerra-Rosas, Víctor Iván Sayago-Monreal, José Luis Gónzalez, Fabiola Carlo-Ricartti, Adrián Alvarado-Aguilar, Fernando Guerrero-Romero, Martha Rodríguez-Morán, Claudia I. Gamboa-Gómez

**Affiliations:** 1 Departamento de Ingenierías Química y Bioquímica, Tecnológico Nacional de México-Instituto Tecnológico de Durango, Felipe Pescador 1830 Oriente, Durango 34080, Mexico; jmorales@itdurango.edu.mx (J.M.-C.); m.guerra@itdurango.edu.mx (M.I.G.-R.); victor_sayago95@hotmail.com (V.I.S.-M.); 2 Centro Estatal de Cancerología, Secretaria de Salud Durango, Av. 5 de Febrero esq. Antonio Norman Fuentes S/N. Zona Centro, Durango 34000, Mexico; ljbarraganz@gmail.com; 3 Departamento de Patología, Hospital General de Zona No. 1, Instituto Mexicano del Seguro Social, Canoas S/N, Durango 34067, Mexico; jlgpatologia@yahoo.com.mx; 4 Unidad de Investigación Biomédica del Instituto Mexicano del Seguro Social, Canoas 100, Durango 34067, Mexico; fabysrc@hotmail.com (F.C.-R.); alvaraguiadrian95@gmail.com (A.A.-A.); guerrero.romero@gmail.com (F.G.-R.)

**Keywords:** husk extracts, maceration, ultrasound-assisted extraction, obesity, hyperglycemia, insulin resistance, hepatic steatosis, oxidative stress

## Abstract

**Background/Objectives**: Plants and fruits of *Physalis philadelphica* Lam. Solanacea are commonly used in traditional medicine to improve some illnesses such as diabetes, in North and Central American countries. The aim was to evaluate the effects of aqueous maceration (He-M) and ultrasound-assisted (He-US) extracts of *P*. *philadelphica* husk on hyperglycemia, insulin resistance, hepatic steatosis, and oxidative stress in obese rats. **Methods:** The effects of husk extracts on carbohydrate and lipid absorption were evaluated using oral starch and lipid tolerance tests in healthy male Wistar rats. Obesity was then induced using a high-fructose and saturated fat diet, followed by 16 weeks of extract administration. **Results**: He-US significantly reduced the postprandial glycemic spike, while both extracts lowered serum triglyceride levels (~50%) following lipid loading, compared with the negative control. In obese rats, both extracts reduced body weight gain (~10%) and lowered fasting glucose levels (22% for He-M and 15% for He-US), compared with the obese control. He-US also reduced insulin levels (~32%), insulin resistance (~53%), and free fatty acids (~52%), while He-M improved hepatic steatosis and reduced liver triglycerides (~26%). Both extracts reduced hepatic nitrite levels, although only He-M significantly decreased lipid peroxidation (~32%). Additionally, both treatments enhanced hepatic antioxidant enzyme activity. **Conclusions**: Husk extracts exerted beneficial effects on hyperglycemia, insulin resistance, hepatic steatosis, and oxidative stress markers in obese rats.

## 1. Introduction

Obesity is characterized by excessive fat accumulation, particularly in the abdominal (visceral) region, and it is widely recognized as an insulin-resistant condition associated with a higher prevalence of glucose and lipid metabolism abnormalities, hypertension, cardiovascular disease, stroke, and non-alcoholic fatty liver disease [1,2,3]. The expansion of adipose tissue due to visceral fat accumulation facilitates macrophage infiltration and promotes excessive lipolysis, leading to elevated circulating free fatty acids (FFAs). This increase in serum FFAs disrupts glucose metabolism, induces insulin resistance (IR), and exacerbates hepatic gluconeogenesis and triglyceride (TG) production [4,5], while also impairing glucose uptake in peripheral tissues [6]. Moreover, the chronic release of FFAs stimulates the production of nitric oxide (NO), which contributes to the degradation of insulin receptor substrate 1 (IRS-1), further aggravating IR [7]. NO is highly reactive with free radicals, especially hydroxyl radicals, leading to the formation of peroxynitrite, a potent inducer of oxidative stress [8]. Additionally, obesity is associated with lipid peroxidation and impaired antioxidant defenses, both of which further intensify oxidative stress [1]. Obesity is also frequently associated with varying degrees of hepatic steatosis, characterized by excessive lipid accumulation in the liver and histological evidence of lobular inflammation [9]. The development of hepatic steatosis is tightly regulated by multiple factors, including altered lipid uptake, reduced fatty acid β-oxidation, and increased de novo lipogenesis [10].

Although pharmacological interventions for obesity have demonstrated therapeutic efficacy, their clinical utility is often limited by adverse effects ranging from mild to severe [11]. This has prompted growing interest in alternative strategies, particularly those based on nutritional interventions aimed at preventing or attenuating the metabolic dysregulation associated with obesity. Among these, natural products enriched in bioactive phytochemicals are gaining attention as potential adjuncts or alternatives to conventional therapies [12]. In this context, members of the *Physalis* L., Solanaceae genus have been investigated for their therapeutic potential due to their well-documented antioxidant and anti-inflammatory properties. For example, Lee et al. [13] demonstrated that oral administration of *Physalis alkekengi* Linn. Solanaceae extract (300 mg/kg body weight) significantly attenuated body weight gain and reduced serum glucose levels in vivo. More recently, Moussa et al. [14] reported that treatment with *Physalis peruviana* L. Solanaceae extract ameliorated several obesity-associated pathological features, including adipose tissue hypertrophy, hepatic lipid accumulation, oxidative stress, inflammatory infiltration, hepatic fibrosis, and metabolic syndrome in rats subjected to a high-fat diet. With respect to native species from Mexico and Central America, our group previously reported that *Physalis ixocarpa* Brot. ex Hornem., Solanaceae extract exerts both hypoglycemic and antioxidant effects in in vitro and in vivo models [15]. Among the approximately 70 *Physalis* species distributed in Mexico—most of which are endemic [16]—*P*. *ixocarpa* and *Physalis philadelphica* Lam., Solanaceae are of agronomic and nutritional importance. These species are widely consumed in Central America and the United States, typically as fresh fruits known regionally as “tomate verde” (green tomato), “tomate de cáscara” (husk tomato), or “tomatillo” [17,18]. The fruit is enclosed in a papery calyx or husk, which serves as a natural protective barrier against abiotic stress, phytopathogens, and herbivores [19]. The protective function of the husk has been linked to its phytochemical composition, particularly its content of polyphenolic compounds with antioxidant and repellent activities [20]. In traditional medicine, husk extracts have been used for the treatment of various ailments, including respiratory and metabolic disorders such as cough, fever, inflammation, and diabetes [21,22,23]. Despite its ethnopharmacological relevance, the husk is currently regarded as an agro-industrial byproduct or waste [24], underscoring the need to re-evaluate its potential for functional food and pharmaceutical applications.

In the phytochemical discovery process from natural products, extraction constitutes a critical and foundational step. The composition and quality of the final extract are significantly influenced by the extraction technique employed [25]. The efficiency and selectivity of plant-derived bioactive compound recovery depend on multiple parameters, including pressure, temperature, solvent polarity, extraction time, and the physicochemical characteristics of the plant matrix [26]. For example, traditional maceration techniques—such as cold, thermal, and enzymatic methods—are widely considered cost-effective and versatile, primarily due to their reliance on simple and readily available equipment [27].

Despite these advantages, such methods exhibit several limitations, including prolonged extraction times, high solvent consumption, low extraction yields, and limited efficiency [28]. Furthermore, they often lack selectivity toward specific bioactive compounds. Consequently, alternative extraction strategies have been explored, with ultrasound-assisted extraction emerging as a promising approach [29]. The ultrasound-assisted extraction leverages the cavitation phenomenon, which is induced by ultrasound frequencies ranging from 20 to 100 kHz. These frequencies generate gas bubbles within the extraction medium, which subsequently collapse, producing intense physical effects—such as microstreaming, microjets, and shock waves—as well as localized chemical reactions. This combination of effects disrupts cellular structures, thereby enhancing the release of intracellular bioactive compounds [30]. Given these mechanistic advantages, ultrasound-based technologies represent a viable and sustainable alternative for improving plant matrix extraction. They offer reduced energy and solvent requirements, improved extraction efficiency, and higher yields, while being more environmentally benign compared to conventional methods [30,31].

The rationale for comparing aqueous maceration and ultrasound-assisted extraction methods stems from our previous findings in *P*. *ixocarpa*, where we reported phytochemical profile variations between extracts obtained through these two techniques, as well as differences in their biological effects in acute settings. These observations suggest that the extraction method can critically influence the composition and activity of the resulting extracts. Building on this evidence, we hypothesized that applying maceration or ultrasound-assisted extraction to *P*. *philadelphica* husk could similarly affect its biological efficacy, particularly under chronic conditions. Accordingly, the objective of this is to evaluate the effects of aqueous maceration and ultrasound-assisted extracts of *P*. *philadelphica* husk on hyperglycemia, hyperlipidemia, insulin resistance, hepatic steatosis, and oxidative stress markers in a diet-induced obesity rat model.

## 2. Results

### 2.1. PH Values, Yields, and Chemical Characterization

The pH values of *P. philadelphica* husk extracts were ~6.0. The yield of husk extract obtained after maceration (He-M) was ~7%, which is equivalent to 13.3 mg of lyophilized extract per mL of aqueous extract, whereas the husk extract obtained by ultrasound (He-US) yield was ~8%, which is equivalent to 15.9 mg of lyophilized extract per mL of aqueous extract.

**Figure 1:**
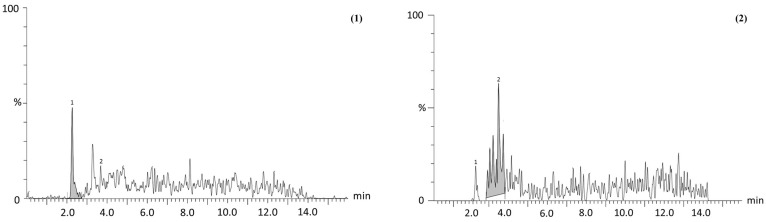
Representative chromatogram of two identified compounds, (**1**) protocatechuic acid and (**2**) chlorogenic acid, from *Physalis philadelphica* husk extracts. Profiles correspond to (A) maceration extract (He-M) and (B) ultrasound extract (He-US). Chromatographic separation was performed by ultra-performance liquid chromatography (UPLC) using a Waters Acquity system. Detection and quantification were achieved with reference standards to confirm retention times, *m*/*z* values, and MS/MS transitions, with analyses carried out in multiple reaction monitoring (MRM) mode.

Regarding chemical characterization, similar bioactive compounds were identified and quantified in both *P*. *philadelphica* husk extracts; however, marked differences in their concentrations were observed. For instance, the He-M exhibited significantly higher levels of protocatechuic acid (1.8-fold), ferulic acid (3.5-fold), and quercetin (2.6-fold) compared to the He-US. In contrast, He-US showed elevated concentrations of gallic acid (1.36-fold), caffeic acid (1.1-fold), chlorogenic acid (8-fold), kaempferol (2-fold), and rutin (85-fold) relative to He-M (Figure 1 and Table 1).

### 2.2. Carbohydrate and Lipid Absorption Assessment

Thirty minutes after starch administration, the oral starch tolerance test (OSTT) showed that the negative control group experienced a marked increase in blood glucose levels (160 mg/dL), whereas the positive control group (acarbose) exhibited a 32% reduction in the glycemic spike. In the treated groups, glucose levels remained slightly above those of the positive control (~11%). After 60 min of starch administration, the He-M group showed no significant differences compared with the negative control, whereas the He-US group exhibited values similar to those of the positive control.

**Figure 2:**
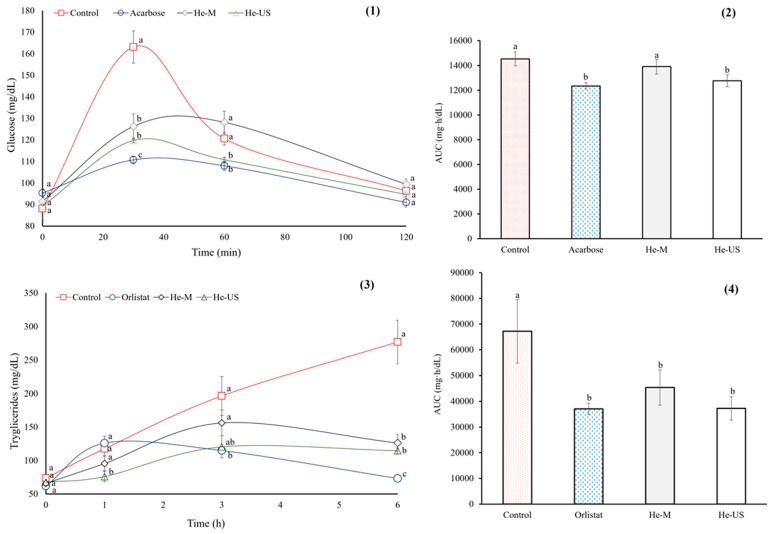
OSTT (**1**), and area under time curve for OSTT (**2**), OLTT (**3**), and area under time curve for OLTT (**4**) of healthy rats that were administered extract obtained after maceration (He-M) and (**2**) husk extracts obtained by ultrasound (He-US). Acarbose and Orlistat^®^ were used as positive controls. Values are expressed as mean ± standard error. Different letters in each subfigure indicate significant differences (*p* < 0.05) between study groups (*n* = 8). Significance was determined using one-way ANOVA followed by Tukey’s post hoc test.

Finally, all groups returned to normal glucose levels after 120 min of starch administration. In this context, the area under time curve (AUC) was similar across all groups (Figure 2).

The oral lipid tolerance test (OLTT) and AUC results are depicted in Figure 2. Both *P*. *philadelphica* husk extracts significantly reduced the TG concentrations as compared with the negative control group, decreasing by ~50% six hours after lipid administration. However, Orlistat^®^ had a greater effect than both *P*. *philadelphica* husk extracts (~73%). Additionally, the AUC was similar for both treated groups and the Orlistat^®^ group.

### 2.3. Effects of P. philadelphica Husk Extracts on Body Weight Gain, Lipid Profile, Fasting Glucose, Insulin Levels, and Insulin Resistance in Obese Rats

**Figure 3:**
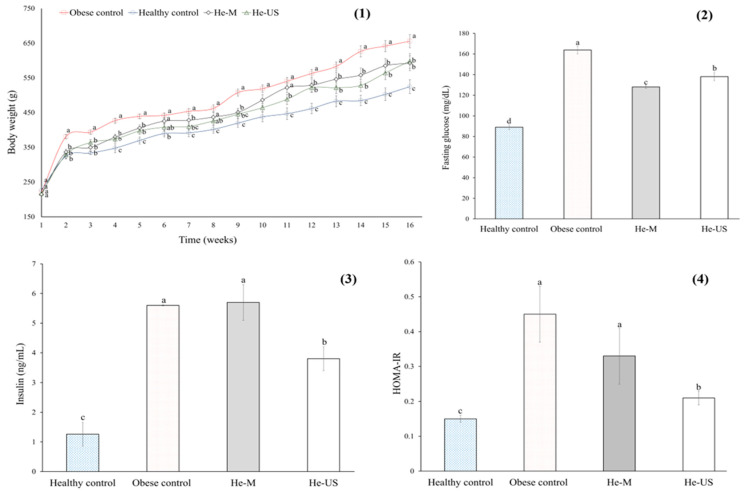
Body weight (**1**), fasting glucose (**2**), insulin (**3**), and HOMA-IR (**4**) values of obese rats that received a *Physalis philadelphica* extract obtained after maceration (He-M) and husk extract obtained by ultrasound (He-US). Values are mean ± standard error. Different letters in each subfigure indicate significant differences (*p* < 0.05) between study groups (*n* = 8). Significance was determined using one-way ANOVA followed by Tukey’s post hoc test.

After 16 weeks on an obesogenic diet (Table 2), obese rats exhibited a 20% increase in body weight gain compared to healthy controls. In contrast, obese rats administered either He-M or He-US showed a significant reduction in weight gain (~10%) compared to the obese control group (Figure 3).

Regarding the lipid profile, after 16 weeks on an obesogenic diet, obese control rats exhibited increased serum levels of TG (~9%), FFAs (~58%), total cholesterol (TC) (~62%), and high-density lipoprotein (HDL-c) (~6%) compared to healthy controls (Table 3). In contrast, rats treated with *P*. *philadelphica* husk extracts showed a significant reduction in FFAs (38% and 52% for He-M and He-US, respectively) and TC (~63% for both treatment groups) compared to obese controls.

In terms of glucose metabolism, obese rats exhibited elevated levels of fasting serum glucose (45%), insulin (78%), and Homeostatic Model Assessment (HOMA-IR) (64%) compared to healthy controls. In contrast, rats treated with *P*. *philadelphica* husk extracts showed a significant reduction in fasting glucose levels (~22% for He-M and ~15% for He-US) (Figure 3). Moreover, treatment with He-US resulted in a significant decrease in serum insulin levels (32%) and HOMA-IR (53%) compared to obese controls.

### 2.4. Effects of Physalis philadelphica Husk Extracts on Steatosis and Hepatic Triglyceride Content in Obese Rats

**Figure 4:**
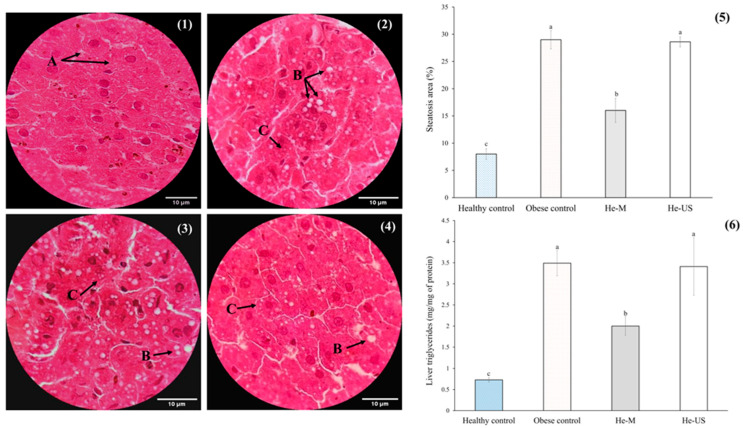
Representative histological sections of liver tissue (H&E staining, 100X; scale bar = 10 µm) from (**1**) healthy control rats, (**2**) obese control rats, (**3**) rats administered husk extract obtained by maceration (He-M), and (**4**) rats administered husk extract obtained by ultrasound (He-US). Hepatocytes (A), macrovesicular steatosis (B), and microvesicular steatosis (C) are indicated. Additionally, (**5**) shows the steatosis area percentage and (**6**) the hepatic triglyceride content. Values are expressed as means ± standard error. Different letters in each subfigure between columns indicate significant differences (*p* < 0.05) between study groups (*n* = 8). Significance was determined using one-way ANOVA followed by Tukey’s post hoc test.

The results related to hepatic steatosis are shown in Figure 4. Compared to the healthy control group, the obese control group exhibited lipid droplet accumulation in the hepatic tissue, indicative of grade 1 steatosis. These findings suggest that macro and microvesicular steatosis, excluding other white regions, occupied approximately 30% of the hepatic parenchyma. Regarding the steatosis area in the treated groups, He-US showed a pattern similar to the obese control group, whereas He-M significantly reduced the steatosis area by approximately 26% compared to both the obese control and He-US groups. Consistently, a significant reduction in hepatic TG content was observed only in the He-M-treated group, which showed a ~42% decrease relative to both comparison groups.

### 2.5. Effects of Physalis philadelphica Husk Extracts on Oxidative Stress Markers in Obese Rats

#### 2.5.1. Nitric Oxide End Products (NO_end-PD_) and Lipid Peroxidation

The results of NO_end-PD_ and lipid peroxidation are shown in Table 4. Compared to healthy rats, the obese control group showed increased concentrations of both NO_end-PD_ (by 59% in serum and 49% in liver) and malondialdehyde (MDA) (by 27% in serum and 63% in liver). In contrast, both treated groups showed a reduction in liver NO_end-PD_ levels (20% for He-M and 33% for He-US), whereas a significant reduction in the liver (32%) was observed only in rats administered He-M.

#### 2.5.2. Antioxidant Enzyme Activity

**Figure 5:**
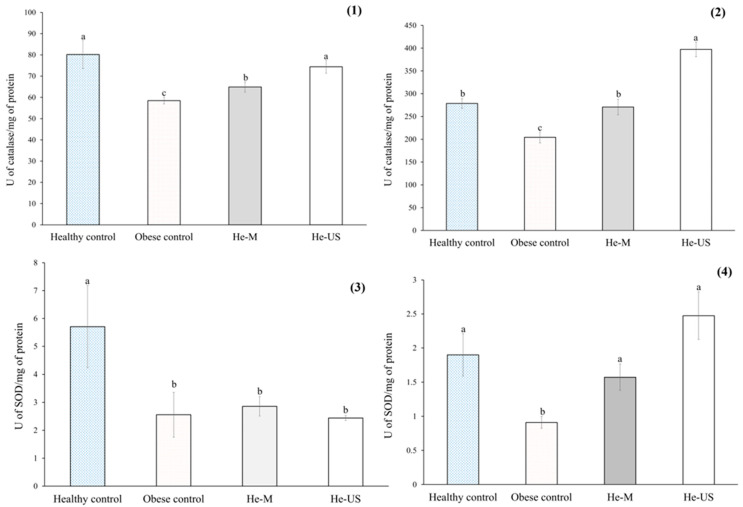
Catalase activity in (**1**) serum and (**2**) liver, and superoxide dismutase (SOD) activity in (**3**) serum and (**4**) liver of obese rats administered *Physalis philadelphica* husk extracts obtained by maceration (He-M) or ultrasound (He-US). Values are expressed as means ± standard error. Different letters between columns in each subfigure indicate significant differences (*p* < 0.05) between study groups (*n* = 8). Significance was determined using one-way ANOVA followed by Tukey’s post hoc test.

The results of antioxidant enzyme activity are presented in Figure 5. Compared to healthy rats, the obese control group exhibited a significant reduction in catalase (CAT) activity—27% in serum and 26% in liver. A similar trend was observed for superoxide dismutase (SOD), with decreases of 55% in serum and 52% in liver. In contrast, rats treated with He-M showed a significant increase in hepatic CAT activity (~32%), while those treated with He-US exhibited increased CAT activity in both serum (27%) and liver (94%) compared to the obese control group. Regarding SOD activity, both treatment groups demonstrated significant increases only in the liver—1.7-fold for He-M and 2.7-fold for He-US—relative to obese controls.

## 3. Discussion

Our findings demonstrate that *P*. *philadelphica* husk extracts exert beneficial effects on hyperglycemia, hyperlipidemia, insulin resistance, hepatic steatosis, and oxidative stress markers in obese rats. These effects were influenced by the extraction method employed. In this regard, we selected two extraction methods to compare a conventional approach (maceration) with a modern technique (ultrasound-assisted extraction, US). Maceration is widely employed for plant extracts due to its simplicity, efficiency, and broad applicability, whereas ultrasound enhances solvent penetration into plant particles, operates at lower extraction temperatures, and requires shorter extraction times [27]. Nevertheless, the use of organic solvents raises concerns about residual toxicity and their suitability for chronic consumption. For this reason, although alternative extraction strategies may broaden the phytochemical profile and enhance the biological potential of *Physalis* husk extracts, we focused on aqueous extracts to ensure safety and long-term relevance. Future investigations should systematically compare different solvent systems and extraction conditions to clarify their influence on both yield and biological efficacy.

In the present study, despite both extracts containing similar classes of bioactive compounds, differences in their concentrations were evident. Specifically, the He-M extract exhibited higher levels of phenolic acids such as protocatechuic and ferulic acids, together with the flavonoid quercetin. In contrast, the He-US extract was enriched in chlorogenic acid, kaempferol, and rutin, resulting in an overall 8% higher concentration of bioactive compounds compared with He-M. These findings are consistent with previous reports showing that ultrasound-based technologies enhance extraction efficiency and yield [30,31]. Such phytochemical differences, both in qualitative composition and quantitative concentration, are likely to contribute to the distinct biological activities observed, particularly in the He-US extract. For example, chlorogenic acid has been shown to reduce body fat accumulation in high-fat diet-induced obese mice [32]. Likewise, kaempferol and rutin have been reported to up-regulate GLUT4 expression, enhance glucose uptake, and improve blood glucose control, glucose tolerance, and insulin sensitivity in obese animal models, with kaempferol demonstrating the strongest antidiabetic and anti-obesity effects [33]. These compositional differences may, at least in part, account for the distinct biological activities observed between the extracts.

In the first stage of this study, we evaluated the effects of *P*. *philadelphica* husk extracts on the digestion and absorption of starch and lipids in healthy rats (prior to obesity induction), with the aim of assessing their acute effects and exploring possible mechanisms underlying their hypoglycemic, hypolipidemic, and anti-obesity potential. Our results indicate that both extracts modulate starch and lipid metabolism, resulting in a reduction in postprandial glucose (~21%) and TG spikes (~50%) compared to the obese control group. These effects may be attributed to bioactive compounds present in *P*. *philadelphica* husk extracts, such as caffeic and chlorogenic acid, which have been reported to influence carbohydrate and lipid absorption through several mechanisms, including the suppression of digestive enzymes, inhibition of micelle formation, and bile acid binding. These actions may partially explain the observed beneficial effects [34,35].

Although the effects of *P*. *philadelphica* husk extracts on the digestion and absorption of starch and lipids were less pronounced than those observed with the positive controls (acarbose and Orlistat^®^), it is important to highlight that, despite their efficacy, these pharmacological agents are commonly associated with gastrointestinal side effects. In many regions, such adverse effects limit their widespread use in routine clinical practice [36,37]. These findings support the potential of *P*. *philadelphica* husk extracts as a natural therapeutic alternative with a possibly more favorable safety profile.

After 16 weeks of administering an obesogenic diet, the body weight of obese control rats increased significantly, which represents an increase of 20% compared to healthy male Wistar rats. In contrast, rats treated with either He-M or He-US showed a significant reduction in weight gain (~10%). Although various anti-obesity mechanisms have been reported for the bioactive compounds identified in *P*. *philadelphica* husk extracts [38,39], we partially attribute the inhibition of carbohydrate and lipid digestion to these compounds, as no differences in food or water consumption were observed. However, further studies are needed to elucidate the potential anti-obesity mechanisms.

It has been reported that even modest weight loss (approximately 5–10% of total body weight) can lead to significant improvements in obesity-related complications [40]. In line with this, our findings demonstrate that *P*. *philadelphica* husk extracts significantly reduced fasting glucose levels. Notably, only rats treated with the He-US extract showed a significant reduction in serum insulin levels and HOMA-IR values, suggesting an improvement in insulin sensitivity. Supporting these results, previous studies have shown that extracts from various *Physalis* species, including *P*. *alkekengi* [13], *P*. *peruviana* [14], and *P*. *ixocarpa* [15], a closely related Mexican variety of *P*. *philadelphica* [23], exert hypoglycemic effects. These findings reinforce the potential of *Physalis* husk extracts as a promising natural therapeutic agent for the management of glucose metabolism disorders. The differences observed between the two extracts may be attributed to variations in their phytochemical profiles, particularly in the He-US extract. Notably, He-US contained significantly higher concentrations of chlorogenic acid (8-fold), kaempferol (2-fold), and rutin (85-fold) compared to He-M. These compounds have been previously associated with improvements in insulin resistance. For example, Hsu et al. [41] demonstrated that rutin administration in insulin-resistant mice activated the insulin signaling pathway, resulting in increased GLUT4 translocation and enhanced glucose uptake. More recently, Ihara et al. [42] reported that chlorogenic acid protects pancreatic β-cells by enhancing the expression of insulin receptor substrate 2 through the activation of cAMP response element-binding protein (CREB), thereby promoting downstream insulin signaling.

Our results showed that *P*. *philadelphica* husk extracts significantly reduced FFA concentrations in obese rats, which may be attributed to the presence of bioactive compounds such as ferulic acid, rutin, and quercetin. These compounds have been reported to inhibit hepatic fatty acid synthesis in mice [43] and may also promote lipophagy, thereby providing substrates for mitochondrial β-oxidation through AMPK-signaling activation [44].

The reduction in FFAs observed in the GTH-US-treated group (~52%) may play a key role in the observed improvement in insulin sensitivity. Elevated FFA levels are widely recognized to contribute to insulin resistance through several mechanisms, including the accumulation of TG and diacylglycerol in hepatocytes and myocytes, impaired tyrosine phosphorylation of insulin receptor substrates, and the activation of multiple serine/threonine kinases [42,45]. Therefore, the ability of *P*. *philadelphica* husk extracts to lower FFA levels may partially explain their beneficial effects on insulin resistance.

Although He-US improved circulating FFA concentrations, this effect was not accompanied by changes in hepatic steatosis or liver TG content. In contrast, rats treated with He-M exhibited a marked reduction (50%) in hepatic steatosis, accompanied by a significant decrease in hepatic TG levels. These findings may be attributed to the differing phytochemical profiles of the extracts, particularly the higher concentrations of phenolic acids such as protocatechuic and ferulic acids found in He-M.

Phenolic acids are generally considered to exhibit greater bioavailability than flavonoids, potentially resulting in higher effective concentrations in target tissues [46]. Supporting this, ferulic acid has been reported to be rapidly absorbed from the stomach in its free form and subsequently conjugated in the liver [47]. The mechanisms by which these compounds exert their effects may involve the suppression of de novo lipogenesis via downregulation of the sterol regulatory element-binding protein 1c (SREBP-1c) pathway, as well as the stimulation of fatty acid β-oxidation, likely mediated through activation of AMP-activated protein kinase (AMPK) [48]. These pharmacokinetic and mechanistic properties may help explain the more pronounced hepatic benefits observed in the He-M-treated group. Nonetheless, further studies are warranted to confirm these hypotheses and elucidate the precise molecular pathways involved.

Our findings confirm that the obesity model induced a state of oxidative stress, as evidenced by significant alterations in all evaluated oxidative stress markers in the obese control group compared to the healthy control. In contrast, antioxidant effects were observed in rats treated with either *P*. *philadelphica* husk extract, although the extent and nature of these effects varied depending on the type of extract, the specific oxidative stress marker assessed, and the tissue analyzed. For example, both extract-treated groups showed a reduction in hepatic NO_end-products_, while a significant decrease in liver MDA levels was observed only in the He-M group. Additionally, He-M treatment significantly increased hepatic CAT activity, whereas He-US treatment led to increased CAT activity in both liver and serum. Regarding SOD, both extracts induced a significant increase in liver SOD activity. These differential effects are likely due to variations in the phytochemical composition of each extract, which depend on the extraction method used. Ultrasound-assisted extraction yields higher concentrations of specific bioactive compounds—as we previously mentioned—than conventional maceration, likely due to enhanced mass transfer, improved solvent penetration, and capillary action [49,50]. Furthermore, bioactive compounds, including polyphenols, exert antioxidant effects through multiple mechanisms, including free radical scavenging, inhibition of lipid peroxidation, metal ion chelation, and modulation of antioxidant enzymes [51]. Therefore, variability in oxidative stress markers between treatments was expected.

Additionally, differences between liver and serum antioxidant responses may be attributed to variations in the absorption, bioavailability, and metabolism of these compounds. *P*. *philadelphica* husk contains polyphenols in the form of esters, glycosides, or polymers, which are not absorbed in their native forms and require hydrolysis by intestinal enzymes or the colonic microbiota to become bioavailable [52,53]. Once absorbed, these metabolites undergo conjugation—such as glucuronidation, sulfation, or methylation—primarily in the liver, which may explain the more pronounced antioxidant effects observed in hepatic tissue [54].

Further studies are needed to elucidate the metabolic fate and tissue distribution of these bioactive compounds and to confirm the observed antioxidant mechanisms.

Several limitations deserve to be mentioned: (1) Acute or sub-chronic toxicity tests were not performed. However, this limitation is mitigated by previous reports indicating no toxicity at doses of 100 mg/kg body weight for extracts from other *Physalis* varieties [55]. (2) Only male animals were used, which may limit the extrapolation of results to females due to potential sex-related metabolic differences. However, the use of a single sex is a common initial approach in preclinical studies to reduce biological variability and better characterize treatment effects. (3) Although the extracts were chemically characterized, the specific in vivo pharmacological activities of the individual phenolic acids and flavonoids were not independently validated. Nonetheless, the observed biological effects are supported by previous reports on the bioactivity of these compounds, and the study design focused on the evaluation of the whole extract, reflecting its potential as a phyto-therapeutic agent. Therefore, despite these limitations, the findings remain robust and provide a solid foundation for future studies.

## 4. Materials and Methods

### 4.1. Plant Material and Extracts

*Physalis philadelphica* Lam. husks were collected from mature fruits donated by a local marketplace in “Francisco Villa”, Durango, Mexico. According to the producers, the fruits originated from the 2020 harvest in Aguascalientes.

The taxonomic identification of the fruit was performed by Dr. Socorro González-Elizondo, and voucher specimens (No. 61091) were stored at the Centro Interdisciplinario de Investigación para el Desarrollo Integral Regional del Instituto Politécnico Nacional, Unidad Durango, México (CIIDIR), for future reference. In addition, the plant’s name was checked on http://www.worldfloraonline.org (accessed on 22 February 2022).

Samples were washed and shade-dried at 25 °C. They were then ground to a particle size of approximately 0.7–1.0 mm using a sieve (Standard Testing Sieves, ASTM E11 specification, VWR Scientific, West Chester, USA) and stored until extraction.

### 4.2. Physalis Philadelphica Husk Extract Extraction

For both used methods, the plant material-to-solvent ratio was 1:13 (w/v) according to previous reports about plant and bioactive compounds extracts [56,57]. In this regard, the dried ground samples (15 g) were added into a graduated flask containing distilled water (200 mL).

Maceration extraction was performed following the methodology described by Ma et al. [58]. Briefly, samples were placed in a shaker (WiseCube^®^ Fuzzy Control System WIS-30, DAIH Scientific Co. LTD, Korea) at 150 rpm and maintained at 25 ± 2 °C for 16 h. The resulting extract was filtered twice before analysis.

Ultrasound-assisted extraction was carried out according to the procedure described by Safdar et al. [59], using a Branson Digital Sonifier SFX 250 (Branson Ultrasonic Corporation, Tampico, Mexico). The mixture (plant material and solvent) contained in the flask was treated with an ultrasonic generator probe that had a flat tip with a ½ inch diameter, and the probe was submerged into the solution to a depth of 15 mm. To ensure a constant temperature of 16 ± 2 °C, external water from a thermostatic bath was circulated while the ultrasound pulse was established to 10 s/off and 10 s/on with an amplitude of 70%. The extract was filtered twice before analysis. The total time for extraction was 3 min.

The pH of both extracts was measured using a potentiometer (Beckman Coulter^®^, Inc., Brea, CA, USA). Subsequently, the extracts were lyophilized using a FreeZone Console Freeze Dry System (Labconco, Kansas, USA) and stored in amber containers until further use.

### 4.3. Solid Yields and Chemical Characterization

The yield extraction percentage (Y%) was estimated using (1):

Y% = [W_1_ (g)/W_2_ (g)] [100] (1)

W_1_ is the weight of the corresponding lyophilized extract, and W_2_ is the weight of *P*. *philadelphica* husk.

To perform chemical characterization, a Waters Corporation Acquity UPLC system (Milford, MA, USA) coupled with a tandem Xevo TQ-S triple quadruple mass spectrometer (Waters Corp., Milford, MA, USA) was utilized. The UPLC system included a sample manager operating at a temperature of 5 °C and a quaternary solvent manager. A column of Acquity UPLC BEH C8 with a particle size of 1.7 μm and dimensions of 100 mm × 2.1 mm, maintained at a temperature of 30 °C, was used to identify the bioactive compounds.

The LC system utilized two solvents for the mobile phase, namely, acidified Milli-Q water with 7.5 mM formic acid (Solvent A) and acetonitrile (Solvent B), for the chemical characterization. The gradient program for the mobile phase involved several steps, beginning with an isocratic hold of 5% Solvent B for 0.8 min, followed by an increase to 10% at 1.2 min and an isocratic hold for 0.7 min. Next, the gradient was increased to 15% at 1.9 min and held for 1.5 min, followed by an increase to 21% at 2.4 min and an isocratic hold for 0.3 min. The gradient was then raised to 27% at 5.7 min and held for 2.3 min, followed by a further increase to 50% at 8.0 min, and a linear gradient of 100% solvent B for column washing at 9.0 min. Finally, a linear gradient of 5% solvent B was utilized for column stabilization, lasting until 11.5 min. The LC system was set to a flow rate of 250 μL/min. Negative-mode electrospray ionization (ESI) (Waters Corp., Milford, MA, USA) was used for the analysis, and the following parameters were applied: 2.5 kV capillary voltage, 300 °C desolvation temperature, 150 °C source temperature, 500 L/h desolvation gas flow, 150 L/h cone gas flow, 0.14 mL/min collision gas flow, 5.0 MS mode collision energy, and 20.0 MS/MS mode collision energy. To detect and measure the bioactive compounds, a combination of reference substances with a concentration of 20 ng/μL was utilized to track the retention times, m/z values, and MS/MS transitions. Both the samples and standards were analyzed using the multiple reaction monitoring modes. The analytical software MassLynx v. 4.1 (Waters Corp, Waters Corp., Milford, MA, USA) was employed for system management and data analysis.

The method was validated following the procedure described by International Conference on Harmonization [60] guidelines. Linearity was evaluated by injecting three replicates (20 μL each) of standard solutions containing flavonoids (rutin, quercetin, kaempferol, epicatechin, and naringin) and phenolic acids (gallic, protocatechuic, vanillic, chlorogenic, caffeic, ferulic, and 4,5-dicaffeoylquinic acids), all purchased from Sigma-Aldrich (St. Louis, MO, USA). LC-MS-grade reagents were used for the preparation of standards and samples. Standard solutions were prepared at four concentration levels ranging from 1 to 20 ng/mL. Precision was evaluated through intra-day and inter-day repeatability experiments, involving three injections of each standard solution per day over four consecutive days. Calibration curves were constructed by plotting peak area versus concentrations using the MassLynx 4.1 SCN 8 for Mass Spectrometry Data Analysis software. The limits of detection (LOD) and quantification (LOQ) were estimated experimentally by injecting a series of dilute solutions with known concentrations until the signal-to-noise ratio for the standards reached 3:1 and 10:1, respectively. The specificity of the method was ascertained by analyzing standard compounds and samples.

### 4.4. Experimental Animals

The animal studies were conducted in compliance with the regulations established by the National Institutes of Health [61] and the Norma Oficial Mexicana (NOM-062-ZOO-1999) [62]. Experimental procedures were approved by the Research Committee of the Mexican Social Security Institute (F-CNIC-2023-048).

Male Wistar rats (*n* = 32), aged twelve weeks and weighing 220 ± 20 g (Universidad Autónoma de México, Campus Juriquilla, Querétaro, Mexico) were maintained in an animal laboratory with 12–12 h light–dark cycle and 26 ± 1 °C of temperature. A one-week acclimatization period was established before initiating the experiment. Throughout this acclimatization phase and during the experiment, the rats had unrestricted access to rodent lab chow 5001 (Purina^®^, Québec, Canada).

### 4.5. Carbohydrate and Lipid Absorption Assessment

Before inducing obesity, acute assays were conducted in normal-weight rats to evaluate the effect of the extracts on lipid and carbohydrate digestion and absorption, using the oral starch tolerance test (OSTT) and oral lipid tolerance test (OLTT). Rats were divided into four groups (*n* = 8): (1) negative control, (2) positive control, (3) extract obtained by maceration (He-M), and (4) extract obtained by ultrasound (He-US). During the first phase, OSTT curves were obtained; after a one-week washout period, OLTT curves were obtained for the same groups.

The negative control for OSTT was starch (3 g/kg of body weight), while acarbose (Laboratorios Alpharma, CDMX, Mex.) was the positive control (5 mg/kg of body weight, equivalent to the human dose of 50 mg consumed during a meal).

In the case of OLTT, the negative control was a lipid solution (corn oil/lard, 1:1) (at a dose of 10 mL/kg of body weight), while the positive control used was Orlistat^®^ (Redustat, Laboratorios Liomont, CDMX, Mex.) (6 mg/kg of body weight, equivalent to the human dose of 60 mg consumed during a meal).

Regarding the dose of *P*. *philadelphica* husk extracts used in this study, our group previously demonstrated that both macerated and ultrasound-obtained extracts from *P*. *ixocarpa*, a closely related species within the same family, at a dose of 102 mg/kg body weight (equivalent to approximately 1 g/day in humans), produced hypoglycemic effects [15]. Notably, this dose does not exceed the human equivalent of ~1 g, which is consistent with values used in herbal intervention studies [63]. Based on this evidence, we selected this dose for the present study.

The equivalent doses were calculated using (2) reported by Reagan-Shaw et al. [64]:

HED = [AD (mg/kg)] [animal km/human km](2)

HED represents the human equivalent dose, and AD is the animal dose. Regarding the Km conversion values, these vary among different animal species and humans. For example, the values for adult humans and rats are 37 and 6, respectively.

Under fasting conditions (~8 h), rats were administered either a starch load (for OSTT) or a lipid load (for OLTT), combined with *P*. *philadelphica* husk extracts (He-M or He-US), acarbose, or Orlistat^®^, depending on the group, via intragastric gavage.

Under basal conditions and at 30, 60, and 120 min for OSTT, or at 1, 3, and 6 h for OLTT post-treatment, blood samples were collected from the lateral tail vein (~300 µL) of the rats. Glucose concentrations were measured using a glucometer (Stat Strip^®^ Glucose, Nova Biomedical, Waltham, MA, USA). For TG measurements, serum was separated from the blood samples, and a commercial enzymatic kit (Biosystem Laboratories, Barcelona, Spain) was used according to the manufacturer’s instructions.

The onset time, peak levels, and area under the time curve (AUC) of glucose and TG variation were analyzed to determine the relative rate of carbohydrate or lipid digestion and absorption.

### 4.6. Obesity Induction

For obesity induction in rats, we followed the methodology described by Gamboa-Gómez et al. [65]. The nutrient and caloric content of the obesogenic diet is shown in Table 1. In this regard, the obesogenic diet contains 27% more calories and 70% more lipids than the standard rodent diet.

The experimental groups used for the assessment of carbohydrate and lipid absorption were maintained as follows: (1) healthy control group receiving a standard rodent diet, (2) obese control group receiving an obesogenic diet, (3) group receiving an obesogenic diet plus He-M, and (4) group receiving an obesogenic diet plus He-US. Both extracts were administered via oral gavage at a dose of 102 mg/kg body weight once daily in the morning. Dosages were adjusted daily according to each rat’s body weight to ensure accuracy and account for weight gain during the experiment.

To control for potential stress associated with gavage, both control groups (healthy and obese) received the same volume of distilled water under identical conditions.

The experimental period lasted 16 weeks, during which rats had ad libitum access to food and water. Body weight was measured weekly, and food and water intake was recorded daily.

### 4.7. Blood Sampling and Liver Dissection

At the end of the experimental period, and under fasting conditions (~8 h), the rats were anesthetized with sodium phenobarbital (50 mg/kg body weight) and euthanized by thoracotomy. Blood samples were then collected from the left ventricle. The liver was promptly removed, rinsed with cold, sterile saline solution (~2 °C, 0.9% NaCl, pH 7) (Pisa^®^, Guadalajara, Jalisco, Mexico) to maintain physiological pH, and subsequently dried, weighed, and portioned. One portion was immediately frozen in liquid nitrogen and stored at −80 °C until analysis. The other portion was preserved in 10% formalin for histological assessment. Blood samples were centrifuged at 3000× *g* for 15 min to obtain serum.

### 4.8. Serum Measurements

Serum measurements of fasting glucose, TG, TC, and HDL-c were carried out using commercial assay kits (Biosystem Laboratories, Barcelona, Spain) following the manufacturer’s instructions. All measurements were performed using an automated A15 spectrophotometer (BioSystems S.A., Barcelona, Spain).

FFA levels were assessed following the methodology described by Falholt et al. [66]. Briefly, serum lipids were extracted using a chloroform–heptane–methanol solution (1:1:1), followed by the addition of 50 mM phosphate buffer (pH 7). Results are expressed as µg of palmitic acid equivalents. A diluted standard solution of palmitic acid (SIGMA Co., St. Louis, USA) was used to generate the calibration curve.

### 4.9. Serum Insulin Concentration and Insulin Resistance Assessment

Insulin levels were determined using a rat insulin enzyme-linked immunosorbent assay (ELISA) kit (Millipore, USA) according to the manufacturer’s instructions. A spectrophotometer (Spectronic^®^ 20 GenesysTM, Spectronic Instruments, USA) was used.

For IR assessment, the Homeostatic Model Assessment (HOMA-IR) was calculated as follows (3):

HOMA-IR= [FI × FG /22.5].(3)

where FI corresponds to fasting insulin (µUI/mL) and FG to fasting glucose (mmol/L).

### 4.10. Tissue Homogenate

For the evaluation of hepatic TG content and oxidative stress markers, a tissue homogenate was prepared following the methodology described by Gamboa-Gómez et al. [67]. Briefly, pulverized frozen samples were homogenized in 50 mM phosphate buffer (pH 7.0) containing 0.5 mM EDTA.

For both tissue homogenate and serum, protein concentration was determined using the Bradford method [68]. Briefly, a calibration curve was prepared with albumin as the standard. Under acidic conditions, protein binding to Coomassie dye induced a color change, which was measured spectrophotometrically at 595 nm. Protein concentration was expressed as milligrams of protein per milliliter.

### 4.11. Hepatic TG Content

TG concentration was determined according to the methodology described by Folch et al. [69]. Lipids were extracted from hepatic tissue homogenates using a mixture of chloroform, heptane, and methanol (1:1:1), with 50 mM phosphate buffer (pH 7.0). TG levels were quantified using commercial assay kits (Biosystem Laboratories, Barcelona, Spain), following the manufacturer’s instructions. To account for tissue variability and ensure accurate lipid quantification, results were expressed as μg of TG per mg of protein.

### 4.12. Hepatic Steatosis Evaluation

For steatosis evaluation, samples preserved in formalin were embedded in paraffin, sectioned at 4–5 μm thickness, and stained with hematoxylin and eosin (H&E). Five images per section were captured, with ten fields analyzed per image. Histological evaluation was performed by J.L.G. The severity of hepatic steatosis was assessed using a semi-quantitative scoring system as follows: Grade 0 = < 5% steatosis (no significant fat accumulation), grade 1 (mild) = 5–33% of hepatocytes affected, grade 2 (moderate) = 34–66% of hepatocytes affected, and grade 3 (severe) = > 66% of hepatocytes affected.

### 4.13. Oxidative Stress Assessment

#### 4.13.1. Lipid Peroxidation

Lipid oxidation was estimated through malondialdehyde (MDA) concentration using the method reported by Rocha-Guzmán et al. [70]. Briefly, homogenized tissue or serum samples (0.5 mL) were mixed with 2.0 mL of a solution consisting of thiobarbituric acid (40.5 mL, 0.8%), sodium dodecyl sulfate (13.2 mL, 8.2%), acetic acid (40.5 mL, 20%, pH 3.5 with NaOH 1 M), and distilled water (5.8 mL). Afterwards, samples were incubated 50 min at 96 °C. Then, samples were ice-cooled and mixed with 2 mL of n-butanol. After centrifugation (3000× *g*, 15 min), the absorbance of the supernatant was measured at 532 nm, using a spectrophotometer (Spectronic^®^ 20 GenesysTM, Spectronic Instruments, USA). The results were expressed as micromoles of MDA equivalents/mg of protein. For generating a calibration curve, hydrolyzed 1,3,3, tetraethoxypropane 99% in 200 µL of HCl 39% was used as the MDA standard [71].

#### 4.13.2. End Products of Nitric Oxide (NO_end-PD_)

The methodology described by Sastry et al. [72] with several modifications was used to measure the levels of NO_end-PD_ metabolites (nitrites). Briefly, samples (serum and liver) were deproteinized with ethanol (70%, 1:10) and subjected to a centrifugation process (6700× *g*, 10 min). To reduce nitrate to nitrite, 50 mg/mL of cadmium chloride was added to the samples. Then, samples were centrifuged again (6700× *g*, 5 min), and 100 µL of supernatant was mixed with 100 µL of the Griess reagent (sulphanilamide and N-(1-napthyl) ethylenediamine) and incubated on an orbital shaker in darkness (25 °C, 15 min). The absorbance of the supernatant was measured at 540 nm, using a spectrophotometer (Thermo Scientific Multiskan^®^, USA). The results were expressed as µM/mg of protein. For generating a calibration curve, a diluted standard solution of sodium nitrate was used.

#### 4.13.3. Catalase (CAT) Activity

A modified version of the method described by Sinha et al. [73] was used to determine CAT activity. Briefly, 50 μL of the sample (serum or liver tissue homogenate) was mixed with 500 μL of H_2_O_2_ (0.2 M) and incubated for 3 min at 26 ± 2 °C. Subsequently, 1 mL of potassium dichromate (K_2_Cr_2_O_7_; 5% in acetic acid, 1:3) was added, followed by incubation for 3 min at 80 °C. After cooling, the absorbance of the supernatant was measured at 570 nm using a spectrophotometer (Multiskan^®^, Thermo Scientific, USA). The H_2_O_2_ concentration in the reaction is directly proportional to the formation of dichromate/acetate. A calibration curve was prepared by diluting H_2_O_2_. The amount of enzyme that decomposes 1 μmol of H_2_O_2_ per minute was defined as one unit (U) of CAT activity.

The CAT activity (U/mg of protein) was quantified using (4):

CAT activity = [Δμmol of H_2_O_2_ /(T_1_-V_1_)] [ABS_1_/ABS_2_] [1/P] (4)

where

∆μmol of H_2_O_2_ = H_2_O_2_ (µmol)/blank–H_2_O_2_ (µmol)/reaction;

T_1_= incubation time (3 min);

ABS_1_ = absorbance of H_2_O_2_ at 0.2 M;

ABS_2_ = blank absorbance;

[ABS_1_/ABS_2_] = factor that was considered for any discrepancy in H_2_O_2_ substrate concentration;

P = sample protein content.

#### 4.13.4. Superoxide Dismutase (SOD) Activity

The methodology reported by Ukeda et al. [74], with several modifications, was used for SOD activity evaluation. Briefly, 50 μL of the sample (serum or liver tissue homogenate), xanthine (200 μL, 3 mM), Na_2_CO_3_ (200 μL, 3 mM), and nitro blue tetrazolium chloride (NBT) (100 µL, 1.5 mM) were mixed. The reaction was catalyzed with xanthine oxidase (10 µL, 1U/mL). Over 10 min at 25 °C, the absorbance was recorded (560 nm). The activity of SOD was quantified by the degree of inhibition of NBT reduction by the enzyme, with one unit (U) defined as the amount of enzyme necessary to suppress the reduction in NBT by 50%. Inhibition percentage was used to calculate enzyme activity according to (5) and (6), and the results units were U/mg of protein.

P0 = [(P1 − P2)/P1] [100](5)

SOD activity = [P0/(50%) (V1)] [1/P3](6)

where

P0 = inhibition percentage;

P1 = absorbance of the blank;

P1 = sample absorbance;

P3 = sample protein content.

### 4.14. Statistical Analysis

Data are presented as mean ± standard error (SE) or median (Min–Max). The Shapiro–Wilk test was used to evaluate data distribution. For parametric data, a one-way analysis of variance (ANOVA) with Tukey’s post hoc test was performed. For non-parametric data, the Kruskal–Wallis test was used. The significance level was established with a *p* value < 0.05.

Statistical analyses were conducted using the IBM SPSS Statistics for Windows, version 20 (IBM Corp., Armonk, New York, NY, USA).

## 5. Conclusions

The results of this study demonstrate that *P*. *philadelphica* husk extracts exert beneficial effects on hyperglycemia, insulin resistance, hepatic steatosis, and oxidative stress markers in obese rats. These effects were influenced by the extraction method: the He-M extract was more effective in improving hyperglycemia, reducing hepatic steatosis, and lowering lipid peroxidation in the liver, whereas the He-US extract significantly improved insulin resistance and enhanced antioxidant enzyme activity, particularly in hepatic tissue.

These findings support their potential as alternative natural therapeutic agents for obesity and related metabolic disorders. Future studies should investigate the specific mechanisms underlying these differences and consider the targeted application of each extract based on their respective bioactivities.

## Figures and Tables

**Table 1:** Chemical characterization and determination of detection limits for method validation of *Physalis philadelphica* husk extracts.

No.	Compound	Transitions	RT (min)	LR (ng/mL)	CE (*r^2^*)	LOD (ng/mL)	LOQ (ng/mL)	He-M (ng/mg of Crude Extract)	He-US (ng/mg of Crude Extract)
1	Gallic acid	169.15 > 125.05, 79.07	1.2	0–20	0.9973	0.03	0.09	0.33 ± 0.03 ^a^	0.45 ± 0.02 ^a^
2	Protocatechuic acid	153.1 >109.0, 91.04	2.1	0–24	0.9976	0.07	0.21	28.0 ± 0.60 ^a^	15.0 ± 1.20 ^b^
3	Chlorogenic acid	137.0 > 93.0	3.3	0–20	0.9983	0.3	0.9	3.00 ± 0.10 ^b^	24.0 ± 1.30 ^a^
4	Vanillic acid	167.1 > 123.0	3.7	0–20	0.9640	0.01	0.03	4.10 ± 0.40 ^a^	2.00 ± 0.30 ^b^
5	Caffeic acid	179.1 > 135.08, 89.09	3.9	0–32	0.9986	0.02	0.06	8.00 ± 0.05 ^b^	9.00 ± 0.50 ^a^
6	Epicatechin	289.1 > 245.1	4.3	0–20	0.9995	0.003	0.009	0.03 ± 0.00 ^a^	0.03 ± 0.00 ^a^
7	Feruilic acid	193.2 > 134.0, 178.07	5.7	0–32	0.9949	0.05	0.015	7.50 ± 0.30 ^a^	2.10 ± 0.1 ^b^
8	Rutin	609.2 > 300.2	5.8	0–20	0.9704	0.003	0.01	0.06 ± 0.00 ^b^	5.10 ± 0.20 ^a^
9	Kaempferol-3-O-Glc	447.3 > 255.1	6.7	0–20	0.9947	0.002	0.006	0.20 ± 0.00 ^b^	0.40 ± 0.00 ^a^
10	Quercetin	301.2 > 151.0	8.4	0–20	0.9898	0.001	0.003	4.00 ± 0.40 ^a^	1.50 ± 0.10 ^b^
11	Luteolin	285.2 > 133.0	8.5	0–20	0.9834	0.002	0.006	0.01 ± 0.00 ^a^	0.04 ± 0.00 ^a^
12	Naringenin	271.2 > 151.4, 119.06	9.2	0–20	0.9988	0.001	0.003	Traces	0.04 ± 0.00 ^a^

Data mean ± standard error. Different letters between rows indicate a significant difference between *Physalis philadelphica* husk extracts (*p* ˂ 0.05) by Tukey’s post hoc test. Retention time (RT), correlation coefficient (CE), linear range (LR), limit of detection (LOD), limit of quantitation (LOQ), husk extract obtained after maceration (He-M), and husk extract obtained by ultrasound (He-US).

**Table 2:** Composition of both the obesogenic and standard rodent diets.

* Standard Rodent Diet		Obesogenic Diet	
Nutrients	% *Per* 100 g	Nutrients	% *Per* 100 g
Carbohydrates	70.9	Carbohydrates	60.0
Proteins	13.9	Proteins	23.0
Fat	15.1	Fat	4.5
Saturated fat	3.9	Saturated fat	--
Polyunsaturated fat	1.1	Polyunsaturated fat	--
Monosaturated fat	4.5	Monosaturated fat	--
		Ingredients	g *per* 100 g
		Powdered standard rodent chow	60
		Lard	10
		Fructose	30
Total energy (kcal/100 g)	372.5	Total energy (kcal/100 g)	511.1

* The Rodent Lab Chow 5001 (Purina^®^, Quebec, Canada).

**Table 3:** Serum lipid parameters at the end of experiment of obese rats who received *Physalis philadelphica* husk extracts.

	Healthy Control	Obese Control	He-M	He-US
TG (mg/dL)	140.0± 8.3 ^b^	154.6 ± 5.8 ^a^	158.1 ± 20.2 ^a^	150.8 ± 15.6 ^a^
FFAs (µg/mL)	24.83 ± 4.1 ^c^	59.3 ± 2.8 ^a^	36.5 ± 3.8 ^b^	28.3 ± 2.8 ^c^
TC (mg/dL)	68.1 ± 3.5 ^b^	180.6 ± 2.3 ^a^	65.8 ± 4.2 ^b^	68.1 ± 3.5 ^b^
HDL-c (mg/dL) *	28.8 (6.0–30.2) ^a^	30 (8.4–33.2) ^a^	24.4 (10.5–30.4) ^a^	23.4 (13–31.9) ^a^

Values are expressed as mean ± standard error, unless otherwise indicated. * Values are presented as median (min–max). Different letters between rows indicate significant differences (*p* < 0.05) between study groups (*n* = 8). Significance was determined using one-way ANOVA followed by Tukey’s post hoc test for parametric data, and the Kruskal–Wallis test was used for non-parametric data. The group received husk extract obtained by maceration (He-M) or ultrasound-assisted extraction (He-US), triglycerides (TG), free fatty acids (FFAs), total cholesterol (TC), and high-density lipoprotein (HDL-c).

**Table 4:** Nitric oxide end products (NO_end-PD_) and malondialdehyde (MDA) concentration in serum and liver of obese rats who received *Physalis philadelphica* husk extracts.

Sample	Serum (µM/mg _protein_)		Liver (µM/mg _protein_)	
	NO_end-PD_	MDA	NO_end-PD_	MDA
Healthy control	0. 21 ± 0.01 ^b^	1.26 ± 0.1 ^b^	0. 23 ± 0.00 ^c^	1.06 ± 0.1 ^c^
Obese control	0.52 ± 0.04 ^a^	1.74 ± 0.30 ^a^	0.45 ± 0.02 ^a^	2.83 ± 0.33 ^a^
He-M	0.45 ± 0.05 ^a^	1.61 ± 0.45 ^a^	0.36 ± 0.01 ^b^	1.92 ± 0.13 ^b^
He-US	0.48 ± 0.05 ^a^	2.10 ± 0.21 ^a^	0.30 ± 0.06 ^b^	2.63 ± 0.42 ^a^

Values are means of duplicated determinations ± standard error. Different letters between rows indicate significant differences (*p* < 0.05) between study groups (*n* = 8). Significance was determined using one-way ANOVA followed by Tukey’s post hoc test. The group received husk extract obtained after maceration (He-M) and husk extract obtained by ultrasound (He-US).

## Data Availability

The data presented in this study are available on request from the corresponding author due to ethical restrictions.

## Abbreviations

He-M	Husk extract obtained after maceration
He-US	Husk extract obtained by ultrasound
OSTT	Oral starch tolerance test
OLTT	Oral lipid tolerance test
FFAs	Free fatty acids
CAT	Catalase
SOD	Superoxide dismutase
NO	Nitric oxide
NOend-PD	Nitric oxide end products
IR	Insulin resistance
HOMA-IR	Homeostatic Model Assessment
MDA	Malondialdehyde
TC	Total cholesterol
TG	Triglyceride
